# Triiodothyronine and Protein Malnutrition Could Influence Pulse Wave Velocity in Pre-Dialysis Chronic Kidney Disease Patients

**DOI:** 10.3390/diagnostics13142462

**Published:** 2023-07-24

**Authors:** Crina Claudia Rusu, Ina Kacso, Diana Moldovan, Alina Potra, Dacian Tirinescu, Maria Ticala, Ancuta M. Rotar, Remus Orasan, Cristian Budurea, Andrada Barar, Florin Anton, Ana Valea, Cosmina Ioana Bondor, Madalina Ticolea

**Affiliations:** 1 Department of Nephrology, University of Medicine and Pharmacy “Iuliu Hatieganu” Cluj, 8 Victor Babeș Street, 400012 Cluj-Napoca, Romania; 2 Department of Nephrology, County Emergency Clinical Hospital Cluj, 3-5 Clinicilor Street, 400006 Cluj-Napoca, Romania; 3 Department of Food Science, Faculty of Food Science and Technology, University of Agricultural Sciences and Veterinary Medicine Cluj-Napoca, Calea Manastur 3-5, 400372 Cluj-Napoca, Romania; 4 Nefromed Dialysis Center, 40 Ana Aslan Street, 400528 Cluj-Napoca, Romania; 5 Department of Cardiology, University of Medicine and Pharmacy “Iuliu Hatieganu” Cluj, 8 Victor Babeș Street, 400012 Cluj-Napoca, Romania; 6 Department of Endocrinology, University of Medicine and Pharmacy “Iuliu Hatieganu” Cluj, 8 Victor Babeș Street, 400012 Cluj-Napoca, Romania; 7 Department of Medical Informatics and Biostatistics, University of Medicine and Pharmacy “Iuliu Hatieganu” Cluj, 6 Pasteur Street, 400349 Cluj-Napoca, Romania

**Keywords:** pulse wave velocity, chronic kidney disease, malnutrition, inflammation triiodothyronine, prolactin

## Abstract

Cardiovascular diseases (CVD) are the first cause of chronic kidney disease (CKD) mortality. For personalized improved medicine, detecting correctable markers of CVD can be considered a priority. The aim of this study was the evaluation of the impact of nutritional, hormonal and inflammatory markers on brachial-ankle Pulse Wave Velocity (PWV) in pre-dialysis CKD patients. A cross-sectional observational study was conducted on 68 pre-dialysis CKD patients (median age of 69 years, 41.2% with diabetes mellitus, 52.9% male). Laboratory data were collected, including levels of prolactin, triiodothyronine, TGF α, IL-6, and IL-1β. The high values of brachial-ankle PWV were associated with reduced muscle mass (*p* = 0.001, r = −0.44), low levels of total cholesterol (*p* = 0.04, r = −0.26), triglycerides (*p* = 0.03, r = −0.31), triiodothyronine (*p* = 0.04, r = −0.24), and prolactin (*p* = 0.02, r = −0.27). High PWV was associated with advanced age (*p* < 0.001, r = 0.19). In the multivariate analysis, reduced muscle mass (*p* = 0.018), low levels of triiodothyronine (*p* = 0.002), and triglycerides (*p* = 0.049) were significant predictors of PWV, but age (*p* < 0.001) remained an important factor. In conclusion, reduced triiodothyronine together with markers of malnutrition and age were associated with PWV in pre-dialysis CKD patients.

## 1. Introduction

High cardiovascular risk in chronic kidney disease (CKD) patients is associated with accelerated atherosclerosis, endothelial dysfunction, and arterial stiffness (AS) and it has major consequences on survival and quality of life. Arterial stiffness is a negative prognostic factor for CKD progression [1] and for associated cardiovascular diseases, contributing to the increase in medical services costs [2,3,4]. Previous studies have suggested that therapeutic modification of AS can improve cardiovascular mortality in CKD [5,6]. AS was influenced by angiotensin-converting enzyme (ACE) inhibitors/angiotensin II receptor blockers (ARB), vitamin D in the pre-dialysis CKD patients [6,7], and by hypotensive medication combined with the reduction of calcium in the dialysis solution in hemodialysis patients [8]. A relationship between increased AS and declining kidney function was shown [8].

According to experts, the PWV remains a standard parameter for the assessment of AS [9,10]. There are different devices for measuring PWV, based on tonometry, oscillometry, and magnetic resonance [11].

AS is characterized by chronic structural modifications in the arterial wall expressed by elastin fragmentation and media calcification, but molecular changes in the intimal layer may also occur through the atherosclerotic inflammatory process. [12,13].

Hyperphosphatemia, the fluctuations of calcium such as hyper and hypocalcemia, hyperparathyroidism, the reduction of alpha Klotho, and the increase in FGF-23 are the main determinants of AS in CKD. Classic cardiovascular risk factors also intervene: hypertension, obesity, dyslipidemia, advanced age (through decreased endothelium nitric oxide availability and increased production of vasoconstrictors), diabetes mellitus (DM), and hyperuricemia [10,11,12]. In addition, advanced glycation end products (AGEs) that accumulate in CKD activate Nuclear Factor Kappa B, favoring the activation of the vascular inflammatory cascade and promoting the vessel’s stiffening by stimulating fibrosis and proliferation of the vascular smooth muscle cells [14,15]. There was also an association of serum glucose concentrations with PWV, independent of the diabetic status [16].

In fact, AS in CKD is based on an enormously increased cardiovascular risk due to, on the one hand, the additional cardiovascular risk factors (oxidative stress, protein malnutrition, alteration of the phospho-calcium balance, etc.) and, on the other hand, due to certain particularities of the classic cardiovascular risk factors such as the appearance of the reverse epidemiology phenomenon [17]. It is well known that malnutrition and inflammation are associated with atherosclerosis (malnutrition inflammation atherosclerosis syndrome) in these patients [18], and it can be a major determinant of vascular stiffness in CKD. There are few data in the literature about the correlation between nutritional markers and PWV in pre-dialysis and dialysis CKD patients. Thus, in a study that evaluated pre-dialysis CKD patients from Korea, it was shown that reduced muscle mass was associated with high brachial-ankle PWV [19]. In addition, another study revealed that hydration status and blood pressure might be major determinants of PWV in hemodialysis patients [20], while in peritoneal dialysis patients a significant association between nutritional markers and PWV was described, suggesting that malnutrition could be the major contributor to vascular dysfunction [21]. It was noted that body mass index (BMI), body fat mass, waist-hip ratio, abdominal circumference, neck circumference, and visceral fat are positively correlated with PWV in the general population [22].

There are also studies that have shown that the hormonal changes occurring in CKD could also influence cardiovascular morbidity and AS. Prolactin is a hormone which is considered as a uremic toxin by some authors. It accumulates with loss of renal function, and it is associated with cardiovascular diseases in the general population and CKD population as well [23]. Hyperprolactinemia is implicated in biological processes such as insulin resistance, metabolic syndrome, inflammation modulation, endothelial dysfunction, and lastly, accelerated atherosclerosis [24,25]. A 27% increased risk of cardiovascular events was observed for each 10 ng/mL prolactin elevation in non-dialysis CKD patients [26].

The presence of subclinical hypothyroidism was also recorded in CKD. It was associated with general mortality in advanced CKD [27]. Low triiodothyronine levels are the most common laboratory finding followed by subclinical hypothyroidism in CKD patients. Hypothyroidism can cause vascular calcification and endothelial damage [28,29].

Currently, it is still not clear how prolactin and triiodothyronine influence cardiovascular diseases in CKD, and if they affect PWV, in fact, what are the most important factors that influence PWV in pre-dialysis CKD patients.

That is why the aim of this study was to evaluate the impact of some inflammatory, nutritional, and hormonal markers on PWV in pre-dialysis CKD patients, and as a second aim in the subgroup of the patients with diabetes.

## 2. Materials and Methods

### 2.1. The Participants

We conducted a cross-sectional observational study on a cohort of pre-dialysis CKD patients. The patients were selected from those admitted to the Department of Nephrology, County Clinical Emergency Hospital Cluj, and taken into this study based on the inclusion and exclusion criteria. All patients provided written informed consent. The study methodology was in accordance with institutional and national research ethical standards and with the 1964 Helsinki Declaration and its subsequent amendments.

Inclusion criteria were the following: patients aged ≥ 18 years, diagnosed with CKD for at least 6 months, defined according to Kidney Disease: Improving Global. Outcomes (KDIGO) guidelines, with estimated glomerular filtration rate (eGFR) less than 60 mL/min, (predialytic stage), having a stable renal function during 3 months prior to study (change in eGFR < 5 mL/min/1.73 m^2^), and no change in medication during the same 3 months.

The exclusion criteria were the following: cancer patients with a life expectancy < 6 months, acute inflammatory diseases, terminal neoplasia, hepatitis viral infection, and any other chronic or acute diseases that required changes in treatment during 3 months prior to study.

The patient’s clinical data: age, weight, height, systolic blood pressure (SBP), and diastolic blood pressure (DBP), comorbidities (diabetes, hypertension) and the medication data were registered. The diagnosis of hypertension was established on the basis of BP values, namely SBP/DBP ≥ 140/90 mmHg as well as on the basis of the use of hypotensive drugs. We calculated pulse pressure (PP) as the difference between the SBP and DBP.

### 2.2. Evaluation of Anthropometric Parameters

In addition to body mass index (BMI), nutritional status was assessed by bioimpedance using the Body Composition Monitor, a certified device (manufacturer by Fresenius Medical Care, Bad Homburg, Germany) which provided body composition as follows: lean tissue mass (LTM) (kg), and adipose tissue mass (ATM) (kg) [30].

### 2.3. Laboratory Parameters

Blood samples were collected in the morning after 8 h fasting. Serum electrolytes, albumin, creatinine, lipid profile, inflammatory markers, intact parathormone (iPTH) and the medular response (hemoglobin and white blood cells) were determined. Serum IL-6, IL-1β, TNF-α, prolactin and triiodothyronine were determined by enzyme-linked immunosorbent assay (ELISA) using commercially available kits (R & D System, Minneapolis, MN, USA). The minimum detection limit for TNF-α was 15.6 pg/mL, for IL-6–3.2 pg/mL, for IL-1β–10.2 pg/mL, for prolactin 1.5 ng/mL, and for triiodothyronine < 0.1 ng/mL). Low-density lipoprotein-cholesterol (LDL-cholesterol) was calculated according to the Friedewald formula: LDL cholesterol = total cholesterol-(HDL-cholesterol + triglycerides/5).

### 2.4. Assessment of Arterial Stiffness

Brachial-ankle PWV was evaluated to assess arterial stiffness with the Mobil-O-Graph NG device (Medexpert Ltd., Budapest, Hungary), based on an oscillometric method. The device gave the augmentation pressure, augmentation index, central SBP, central DBP, and PWV. Brachial BP [31] was initially recorded, then the cuff was automatically re-inflated above DBP for approximately 10 s and brachial pulse waves were recorded with a high-fidelity pressure sensor (MPX5050, Freescale Halbleiter Deutschland GmbH, Muenchen, Germany). Brachial BP was used to calibrate the pulse waveform. Finally, the aortic pulse wave form was reconstructed by the software (HMS version 5.1) using an ARCSolver algorithm [32,33]. The aortic pulse wave was decomposed into forward traveling (incident) and backward traveling (reflected) pulse waves for wave separation analysis. PWV was estimated by mathematical models based on the characteristic impedance and age and assuming a three-element Windkessel model [32,33].

### 2.5. Statistical Analysis

Data were presented using different statistical measures depending on the nature of the variables. For normally distributed variables the mean ± standard deviation (SD) was reported. For non-normally distributed variables, median (25th–75th percentile) was used. Nominal variables were expressed as absolute and relative frequencies.

To examine the relationships between quantitative variables, either the Spearman or Pearson coefficient of correlation was employed. Spearman coefficient of correlation was used when the relationship was non-linear or when the outliers were present.

In the multivariate linear regression analysis, PWV was considered the dependent variable. Independent variables included those that showed significant correlation in the univariate analysis and those previously identified in relevant literature as influencing PWV levels were considered. However, SBP was excluded from the model due to multicollinearity.

To compare two groups, different statistical tests were employed based on the nature of the variables. The *t*-test or Mann–Whitney U test for quantitative variables depending on their distribution (normal and non-normal, respectively), while the Chi-square test or Fisher exact test was used for qualitative variables. A *p*-value less than 0.05 was considered statistically significant.

## 3. Results

In this study 80 patients were selected, from which six patients were excluded: four with acute infection, one with acute myocardial infarction, and one with malignancy. Another six patients were excluded due to missing data. Finally, 68 patients remained in the study.

### 3.1. Patients’ Characteristics

The demographical, clinical and laboratory patients’ characteristics are presented in Table 1. In our group, the median (25th, 75th percentile) age was 69 (62.5, 76) years; 41.2% had diabetes and 52.9% were men.

### 3.2. Determinants of Brachial-Ankle PWV

**Figure 1:**
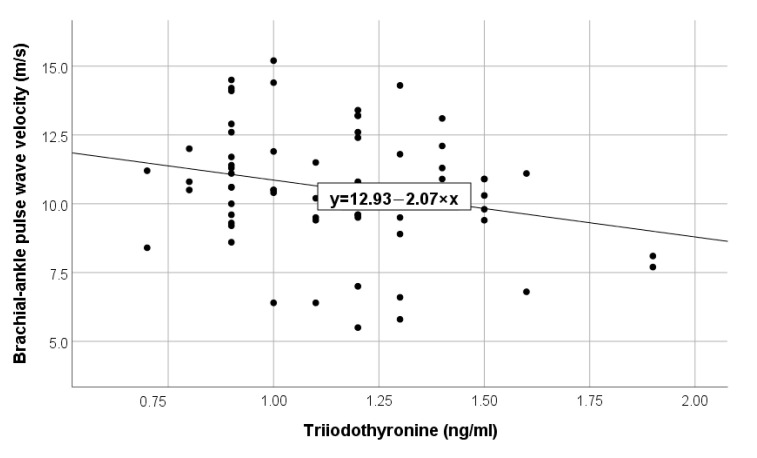
Negative linear correlation between PWV and triiodothyronine in the total group.

**Figure 2:**
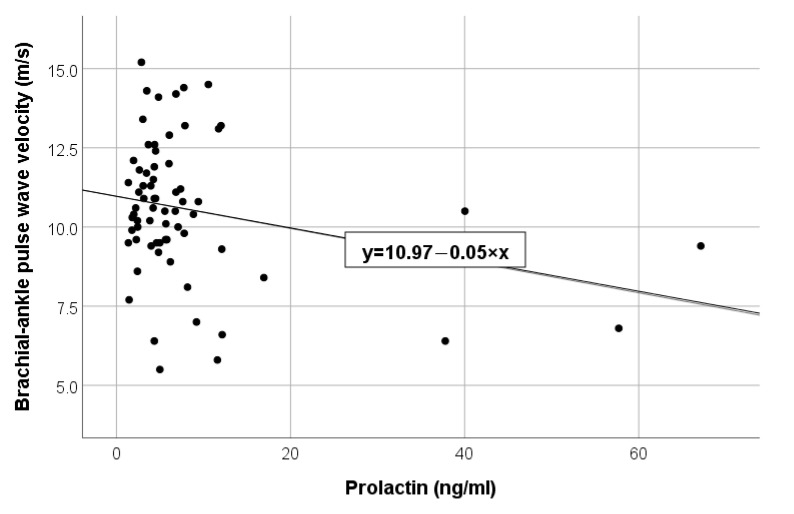
Negative linear correlation between PWV and prolactin in the total group.

In the analysis of correlations, it was observed that high values of brachial-ankle PWV were associated with reduced values of muscle mass (*p* = 0.001, r = −0.45), low levels of total cholesterol (*p* = 0.042, r = −0.26), triglycerides (*p* = 0.023, r = −0.34) and, respectively, low levels of the hormonal engage: triiodothyronine (*p* = 0.04, r = −0.25) (Figure 1) and prolactin (*p* = 0.026, r = −0.27) (Figure 2). Additionally, increasing brachial-ankle PWV was directly associated with high values of SBP (*p* < 0.001, r = 0.56), PP (*p* < 0.001, r = 0.57) and advanced age (*p* < 0.001, r = 0.92), all of these findings are described in Table 2 listed below.

In the multivariate analysis it was noted that muscle mass (*p* = 0.019) and triiodothyronine (*p* = 0.014), PP (*p* = 0.013), and triglycerides (*p* = 0.024), respectively, remained with a significant impact on brachial-ankle PWV, but its strongest determinant was age (*p* < 0.001).

### 3.3. Analysis of the Subgroups

Diabetic vs. non-diabetic patients were analyzed (Table 3). In the DM subgroup there were significantly higher values of SBP (*p* = 0.010) and PP (*p* = 0.009) identified, significantly higher ATM (*p* = 0.032), and significantly higher IL-1β levels (*p* = 0.015).

In the DM subgroup, brachial-ankle PWV was directly correlated with inflammatory markers (TNF alpha *p* = 0.012, r = 0.46; IL-6 *p* = 0.034, r = 0.40), age (*p* < 0.001, r = 0.39) and serum phosphorus (*p* = 0.012, r = 0.39), but not with the eGFR (Table 4).

In the multivariate analysis the parameters which were correlated significantly with brachial-ankle PWV were included in our study, and it was noted that only age (*p* < 0.001) remained statistically significantly associated with PWV.

## 4. Discussion

Age was the strongest determinant of arterial stiffness in the studied group, even though triiodothyronine and prolactin values were also correlated with brachial-ankle PWV. Similar data were published from the CRIC study, in which the worsening of AS with age, the reduction of eGFR, and the increase in PP in CKD were noted [34]. Additionally, in our study, strong correlations of PWV with SBP and PP values were obtained. It is known that hypertension, diabetes mellitus, and CKD are the major determinants of the loss of elasticity and reduced compliance of the vascular wall and, consecutively, increased arterial stiffness. Impaired collagen-elastin ratio, calcification of blood vessels, endothelial dysfunction, increased intima media-thickness, and genetic determinants can produce arterial wall remodeling [35]. All these factors have a prevalence that increases with age.

In addition, it is known that malnutrition is among the risk factors for atherosclerosis and, implicitly, for the increase in AS in CKD. In our study, we remarked that reduced values of muscle mass, therefore protein malnutrition, were associated with the increase of AS. In the longitudinal analysis, in the CRIC study, serum albumin concentration, which is another marker reflecting the protein nutritional status, was predictive of changes in PWV over time [34]. In addition, Harada et al. [36] observed that malnutrition in CKD was a factor associated with vascular calcifications and, consecutively, arterial stiffness, while Cordeiro, in a study, emphasized that another parameter reflecting the nutritional status, the abdominal fat, was associated with coronary artery calcification in non-dialysis dependent CKD patients [37] and then stiffening. In addition, secondary to the reverse epidemiology phenomenon of cardiovascular risk factors in CKD, we noticed that reduced values of lipid markers were associated with increased PWA, not with cardiovascular protection. Therefore, low values of total cholesterol and triglycerides may show a poor nutritional status in this population group.

Regarding the low values of T3 in CKD, they can express a deficit of thyroid function, known in this group of patients, most of the time subclinical (without having a thyroid disease as a substrate) and this could be associated with increased cardiovascular risk. Low values of T3 were associated with AS in our study, consistent with other studies in which FT3, was inversely associated with arterial stiffness in CKD patients [38]. In fact, Klotho synthesis seems to be influenced by the thyroid hormone level [39], and Klotho has a vascular protective effect by reducing vascular calcification. Therefore, the alteration of thyroid hormones in CKD may increase vascular calcifications by reducing the protective effects of Klotho [38]. In addition, overt hypothyroidism has been associated with altered vascular function and altered endothelial-dependent vasodilation [40], partly because of the lack of vasodilatory effect of triiodothyronine (subsequent vasodilation was reported when triiodothyronine increases the NO production by endothelial and smooth muscle cells) [41,42,43]. An increase in central arterial stiffness may be due the overt hypothyroidism as previous studies have shown.

Prolactine was reported to be correlated with PWV. Carrero et al. observed increased prolactin levels in subjects with endothelial dysfunction/stiffness and which further increased the risk of cardiovascular events and mortality [26]. The increase in prolactinoma in CKD is determined by the reduction of its metabolism, by the increased secretion of PRL in the uremic state and by the reduced availability of dopamine in the brain. Secondary to the decrease in dopaminergic activity, there can be an increase in the release of norepinephrine with a negative result on the endothelial function and other organs, favoring myocardial hypertrophy, hypertension, and other cardiovascular diseases [44]. On the other hand, prolactin retention can inhibit the production of gonadotropic hormone, and consequently induce a testosterone deficiency in male patients with CKD, and through this mechanism, atherosclerosis. Prolactin retention was indeed linked to increased intima–media thickness, atherosclerotic plaque occurrence, systemic inflammation, and cardiovascular risk [45,46].

As evidence in our study, not the high values, but the low values of prolactin, a polypeptide hormone, were associated with the increase in PWA and we consider this type of association as an effect of the protein malnutrition present in the patients enrolled in the study. Moreover, in the study by Haring et al., they noted the association of low prolactin values with increased left ventricular mass, these changes only affecting males [47], without finding a clear explanation. Prolactin has 23 kDa and can induce angiogenesis. After proteolytic cleavage, a 5.6–18 kDa, isoform of prolactin, called vasoinhibins, appears with antiangiogenic properties [48]. Thus, the balance between prolactin and vasoinhibins regulates vascular functions [49].

In diabetic patients, PWV is higher than in the general population and promotes an increase in general and cardiovascular mortality [50]. If a diabetic patient has CKD, s/he has also all specific CKD cardiovascular risk factors and PWV increases additionally, with the impact being more significant. In the present study, the analysis of the subgroup of diabetic patients highlighted several aspects. First, age was also the strongest determinant of PWA values. Second, we did not identify significant differences between PWV in diabetics vs. non-diabetics, although we identified several cardiovascular risk markers that were significantly modified in the DM group. Thus, IL-1β (an inflammatory marker) was significantly higher as well as SBP, PP, and adipose tissue mass (expressed by ATM). Third, other inflammatory markers, TNF alpha, and IL-6, as well as phosphorus, a marker of mineral and bone metabolism, were found among the factors significantly associated with the PWA value in the subgroup with DM.

Other studies also reported that inflammatory markers such as fibrinogen and IL-10 were independently associated with PWV [34,51]. Moreover, it is known that micro-inflammation is present in CKD from the early stages and that there is a link between inflammation and atherosclerosis regarding malnutrition (malnutrition inflammation atherosclerosis syndrome). Several possible pathophysiological pathways can explain the association between chronic inflammation and arterial wall disease [14]. Initially, the circulation of inflammatory mediators favors leukocyte migration into the arterial wall [52]. Then, macrophages’ activation by different factors amplifies the inflammatory reaction. This inflammatory cascade then alters the endothelium’s function that interacts and conditions the remodeling of the tunica media, further along with changing the artery’s mechanical properties [53]. Moreover, endothelial cells decrease the usual production of nitric oxide (NO) and increase endothelin (E1), favoring arterial stiffness.

Regarding the connection between phosphorus and AS as noted in our study, it is probably via vascular calcification. In fact, CKD alters hormonal processes that regulate phosphate levels (intestinal absorption, renal excretion by remaining nephrons, bone metabolism modulated by vitamin D, fetuin-A, Klotho, and fibroblast growth factor 23 (FGF-23); all these processes mentioned favoring hyperphosphatemia [54]. Excessive levels of phosphorous and calcium are endogenous minerals capable of stimulating the phenotypic transformation of vascular smooth muscle cells into osteoblast-like cells [55]. Experimental studies indicate that arterial medial calcification-related vascular alterations develop in the early stages of CKD [56].

All these processes initiated in pre-dialysis stages of CKD explain the significant cardiovascular changes detected in dialysis CKD patients [57].

In conclusion, in pre-dialysis CKD patients, age is the strongest determinant of PWV even among diabetic patients. Reduced triiodothyronine and prolactin values are associated with arterial stiffness, while also being markers of malnutrition. Inflammatory markers and hyperphosphatemia influenced PWV in diabetic patients. No variations of PWV were recorded with eGFR or determined by the DM presence. Therefore, we speculate that if we detect and treat the inflammatory syndrome, respectively the malnutrition, the triiodothyronine, and prolactin levels, probably the value of PWV can be influenced. We believe that knowing the factors that influence PWV as a marker of AS, can help to administer a personalized treatment.

The study has some limitations, the first being the relatively small number of included patients, which makes additional studies necessary in order to validate the correlations and associations between PWV and the level of triiodothyronine, prolactin, and inflammatory markers with nutritional status. Secondly, due to the nature of our cross-sectional data, this study was limited in what we can infer about the causality of the results. Thirdly, by design it was an observational study and the conclusions need to be confirmed in the future, possibly by larger prospective studies.

## Figures and Tables

**Table 1:** Characteristics of participants.

Parameter	Group *(n* = 68)
Age (years)	69 (62.5, 76)
Male, *n* (%)	36 (52.9)
Diabetes mellitus ^1^, *n* (%)	28 (41.2)
Hypertension, *n* (%)	60 (89.6)
SBP (mmHg)	144 (126.5, 162)
DBP (mmHg)	87.07 ± 12.42
PP (mmHg)	58 (45.5, 72.5)
eGFR (mL/min/1.73 m^2^)	27 (15, 42)
Body mass index (kg/m^2^)	28.6 (26.4, 30.35)
LTM (kg)	38.25 ± 12.16
ATM (kg)	41.74 ± 13.77
Total cholesterol (mg/dL)	177.49 ± 37.45
LDL-cholesterol (mg/dL)	98.73 ± 28.61
HDL-cholesterol (mg/dL)	43.86 ± 12.94
Triglycerides (mg/dL)	125 (91.5, 166)
Fasting glucose (mg/dL)	103 (92, 131)
Calcium (mg/dL)	9.2 (8.64, 9.69)
Phosphorus (mg/dL)	3.66 (3.14, 4.56)
iPTH (pg/mL)	108.85 (84.85, 227.35)
Alkaline phosphatase (UI/L)	80 (72, 96.5)
Hemoglobin (g/dL)	12.46 ± 2.22
Serum albumin (g/L)	3.89 ± 0.49
hs-C reactive protein (mg/dL)	0.47 (0.23, 1.19)
White blood cells (no./mm^3^)	7625 (6340, 9050)
TNF-α (pg/mL)	4.4 (2.94, 6.8)
IL-6 (pg/mL)	2.44 (1.7, 3.55)
IL-1β (pg/mL)	7.06 (6.45, 12.99)
Prolactin (ng/mL)	4.83 (3.1, 7.76)
Triiodothyronine (ng/mL)	1.2 (0.9, 1.3)
Brachial-ankle PWV (m/s)	10.55 ± 2.17
ACEI/ARB, n (%)	29 (42.6)

^1^ data about 66 patients. Arithmetic mean ± standard deviation; *n*—number of people; no.—number of cell; SBP—systolic blood pressure; DBP—diastolic blood pressure; PP—pulse pressure; eGFR—estimated glomerular filtration rate; LTM—lean tissue mass; ATM—adipose tissue mass; LDL—low-density lipoprotein; HDL—high-density lipoprotein; iPTH—intact parathormone; PWV—pulse wave velocity; ACEI—angiotensin-converting enzyme inhibitors; ARB—angiotensin II receptor blockers.

**Table 2:** The brachial-ankle PWV correlation in the group.

Parameters	r—Coefficient of Correlation	*p*
Age (years)	0.92	<0.001
SBP (mmHg)	0.56	<0.001
PP (mmHg)	0.57	<0.001
Lean tissue mass (kg)	−0.45	0.001
Prolactin (ng/mL)	−0.27	0.026
Triiodothyronine (ng/mL)	−0.25	0.040
Total Cholesterol(mg/dL)	−0.26	0.042
Triglycerides (mg/dL)	−0.34	0.023

SBP—systolic blood pressure, PP—pulse pressure.

**Table 3:** Comparisons between diabetes vs. non-diabetes groups (*n* = 66).

Parameter	Non-Diabetes Subgroup (*n* = 38)	Diabetes Subgroup *(n* = 28)	*p*
Age (years)	68.5 (61, 76)	68 (62.5, 76)	0.689
Male, *n* (%)	17 (45.9)	13 (46.4)	0.969
Hypertension, *n* (%)	34 (89.5)	24 (88.9)	0.940
SBP (mmHg)	138 (121, 156)	150.5 (138.5, 172)	0.010
DBP (mmHg)	82 (76, 98)	89 (80, 97.5)	0.508
PP (mmHg)	49.5 (41, 68)	63 (52, 74)	0.009
eGFR (mL/min/1.73 m^2^)	26 (19, 38)	27.85 (12.5, 47)	0.910
BMI (kg/m^2^)	28.45 (25.5, 29.9)	29.5 (27.1, 31.55)	0.109
LTM (kg)	38.35 ± 12	38.13 ± 12.63	0.948
ATM (kg)	38.27 ± 12.19	46.22 ± 14.62	0.032
Total cholesterol (mg/dL)	185.97 ± 36.06	168.62 ± 36.58	0.072
Triglycerides (mg/dL)	130 (94, 159.5)	124 (85.5, 175.5)	0.961
Calcium (mg/dL)	9.34 (8.67, 9.76)	9.17 (8.78, 9.47)	0.410
Phosphorus (mg/dL)	3.63 (3.13, 4.41)	3.7 (3.3, 5.22)	0.425
TNF-α (pg/mL)	4.6 (3.11, 6.03)	4.02 (2.69, 8.3)	0.678
IL-6 (pg/mL)	2.44 (1.7, 3.27)	2.2 (1.6, 4.36)	0.830
IL-1β (pg/mL)	6.86 (6.38, 9.96)	9.54 (6.79, 19.35)	0.015
Prolactin (ng/mL)	4.98 (3.07, 8.16)	4.66 (3.91, 7.24)	0.912
Triiodothyronine (ng/mL)	1.2 (0.9, 1.3)	1.1 (0.9, 1.35)	0.568
Brachial-ankle PWV (m/s)	10.14 ± 2.42	10.94 ± 1.58	0.135

Arithmetic mean ± standard deviation; SBP—systolic blood pressure; DBP—diastolic blood pressure; PP—pulse pressure; eGFR—estimated glomerular filtration rate; LTM—lean tissue mass; ATM—adipose tissue mass; PWV—pulse wave velocity.

**Table 4:** The brachial-ankle PWV correlation in the subgroup of patients with DM.

Parameters	r—Coefficient of Correlation	*p*
Age (years)	0.89	<0.001
Phosphorus (mg/dL)	−0.51	0.014
eGFR (mL/min/1.73 m^2^)	0.11	0.593
TNF-α (pg/mL)	0.47	0.012
IL-6 (pg/mL)	0.40	0.034

eGFR—estimated glomerular filtration rate.

## Data Availability

Not applicable.
